# Effect of Calcium Hydroxide on the Fracture Resistance of Dentin

**DOI:** 10.6028/jres.116.017

**Published:** 2011-08-01

**Authors:** Evan R. Whitbeck, George D. Quinn, Janet B. Quinn

**Affiliations:** Comprehensive Dentistry Department, Naval Postgraduate Dental School, Bethesda, MD 20889; American Dental Assocoation Foundation, Paffenbarger Research Center, National Institute of Standards and Technology, Gaithersburg, MD 20899

**Keywords:** calcium hydroxide, edge chipping, fracture resistance, human dentin, pulpectomy, teeth

## Abstract

An increased incidence of fracture has been reported in teeth where root canals were treated with calcium hydroxide. Edge chipping is one test used to measure the resistance of brittle materials to fracture. Presently, no studies have reported on edge chipping in teeth. This study evaluated the fracture resistance of human dentin exposed to calcium hydroxide for up to 60 days using the edge chipping method. Twelve recently extracted teeth were divided into a control group and three experimental groups with varying calcium hydroxide exposures. All teeth underwent pulpectomy via standard protocol. It was expected that the edge chip resistance would decrease as a function of exposure, but the results showed the converse. Chip resistance may reflect both the fracture resistance and the hardness of dentin, a quasi brittle material.

## 1. Introduction

Calcium hydroxide has been used for many years in dentistry. Indications for its use include pulp capping to induce dentin bridge formation [1, 2, 3], fostering apical closure of permanent teeth [1, 2, 4, 5, 6], promoting resolution of periapical and resorptive lesions [3, 4, 7, 8] and as an inter-appointment disinfectant in the root canal space [3, 6, 8, 9, 10]. While research has not yet determined the exact mechanism of action, several theories have been proposed [1, 7, 8, 9, 10, 11, 12, 13, 14, 15]. Diffusion of hydroxide ions through dentinal tubules can elevate dentinal pH to as high as 11 in some locations and may contribute to several of its anticipated actions via DNA damage or protein denaturation. Dissolution of necrotic material, neutralization of acid and remineralization of tooth structure via release of calcium ions may also contribute to the beneficial effects of calcium hydroxide.

Despite these clinical successes, reports have surfaced correlating intracanal placement of calcium hydroxide with an increased incidence of tooth fracture, especially in teeth treated prior to full apical closure [2–4]. Researchers have suggested that increased pH alters the strength of the bond between hydroxyapatite and collagen fibrils, induces conformational change in proteoglycan molecules and exerts a proteolytic effect via increased matrix metalloproteinase activity [14, 16, 17]. The exact mechanism by which teeth become more susceptible to fracture is not known with certainty, but may be a combination of these.

A number of investigators have attempted to demonstrate this trend [8, 14, 15, 16, 17,]. These groups, while trying to replicate *in vivo* conditions with whole teeth, introduced significant experimental uncertainty through variations in tooth size, dentin thickness or anatomical defects. Others studied fracture strength [9, 10], but attempted to control experimental variables by preparing standardized specimens. Dentin cylinders or bars milled from bovine or human teeth respectively, were treated with calcium hydroxide and subjected to compressive or shear forces. The latter groups treated prepared test specimens with calcium hydroxide, a methodology bearing little resemblance to everyday clinical practice.

Doyon et al. [3] used an experimental protocol that most resembles clinical procedures. They treated teeth in anatomical form using hand and rotary instrumentation, placed calcium hydroxide and sectioned the roots into disk-shaped specimens which were then compressed with a punch until compressive fracture occurred.

While strength test outcomes may be meaningful, their value may vary with testing methodology. Fracture toughness, or the resistance to crack propagation, is a more inherent property of materials and a better measurement for comparing resistance to fracture [18, 19]. No studies were located that examined the effect of calcium hydroxide on the fracture toughness of dentin.

**Fig. 1:**
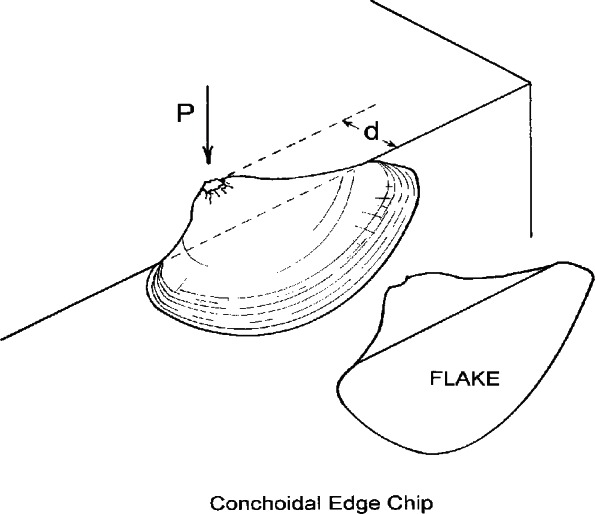
Schematic of the edge chipping test. A stylus or indenter is advanced into a material with a force P until a chip or flake is produced. Chips exhibit little variation in shape between materials, provided that the force applied by the stylus is roughly normal to the surface. Progressively more force is required to produce chips as the distance from the edge, d, is increased.

Edge chipping, which is correlated to fracture toughness for many materials, is a test used to evaluate the resistance of brittle materials to flaking near an edge [20, 21, 22, 23, 24, 25, 26, 27, 28] as shown in Fig. 1. It has been used with dental restorative materials in several studies [29, 30, 31, 32]. Chips or flakes are intentionally formed by advancing an indenter or stylus into a material near an edge. The force required for chip formation is recorded and the thickness of the chip measured. The shape of the chip is relatively independent of the material tested and resembles those seen in fine china and other materials. This test has been used in ceramics, glass and dental materials research but heretofore not with human teeth. For many glasses and ceramics, the force necessary to produce a chip varies linearly with the distance from the edge. The slope of a line through the data describes the susceptibility of the material to edge chipping and has been defined as the *edge toughness*, *T_e_*. The purpose of this *in vitro* pilot study was to evaluate changes in fracture resistance in fully developed human teeth treated with calcium hydroxide using an edge chipping test and to evaluate the suitability of this test.^1^

## 2. Materials and Methods

### 2.1 Materials

Teeth stored in 1 % chloramine-T obtained from an existing store were examined with an operating microscope (Global Surgical Systems, Saint Louis, MO, USA)^2^ and radiographed from a mesio-distal perspective. Exclusion criteria included root caries, root cracks, root resorption or other anatomical defects, teeth with incomplete apical development and those too small to produce the test specimen (about 10 mm in length). Twelve teeth were selected and randomly assigned into four groups each of which contained one incisor, one premolar and one molar. Institutional Review Board (IRB) approval was obtained from the National Naval Medical Center IRB prior to beginning the project.^3^ As extracted tooth specimens used in the project had no identifiable links to human subjects, the research was exempt under 45 CFR part 46.

### 2.2 Methods: Pulpectomies

To simulate clinical conditions, pulpectomies were performed on all teeth in a standardized manner. For each tooth, conventional access cavities were prepared using a #557 carbide bur, a #4 round diamond bur, and an Endo-Z bur (Dentsply Maillefer, York, PA, USA). Canal patency was confirmed by inserting a #10 Flex-O File (Dentsply Maillefer, York, PA, USA) to the apical foramen. Working length was established by subtracting 1 mm from the length measured with the file tip flush to the root surface at the apical foramen. Gates-Glidden burs (Dentsply Maillefer, York, PA, USA), size #4, 3 and 2, were used for shaping of the canal orifices. Profile 0.06 taper rotary files (Dentsply Maillefer, York, PA, USA) were used to shape the canals to 3 mm short of working length. Flex-O files were used to hand instrument the final 3 apical mm. Because sodium hypochlorite alters the dentin properties, sterile saline was selected as the irrigant between files and for the final rinse. All canals were dried with paper points.

The teeth in group 1 served as controls. Following pulpectomy, a cotton pellet was placed and the access cavity closed with a Cavit (3M ESPE, Saint Paul, MN, USA) provisional restoration. For groups 2–4, the canals were dressed with a 50 % (mass fraction) mixture of USP calcium hydroxide powder (Henry Schein, Inc., Melville, NY, USA) and de-ionized, distilled water (Baxter Healthcare Corporation, Deerfield, IL, USA) [9, 33] followed by placement of a cotton pellet and Cavit.

### 2.3 Methods: Edge Chipping

Following pulpectomy treatment, the controls were edge chipped (day 0). Teeth in groups 2, 3, and 4 were subjected to edge chipping on days 15, 30, and 60, respectively. All experimental teeth were stored in a humidor (37 °C) until tested.

**Fig. 2:**
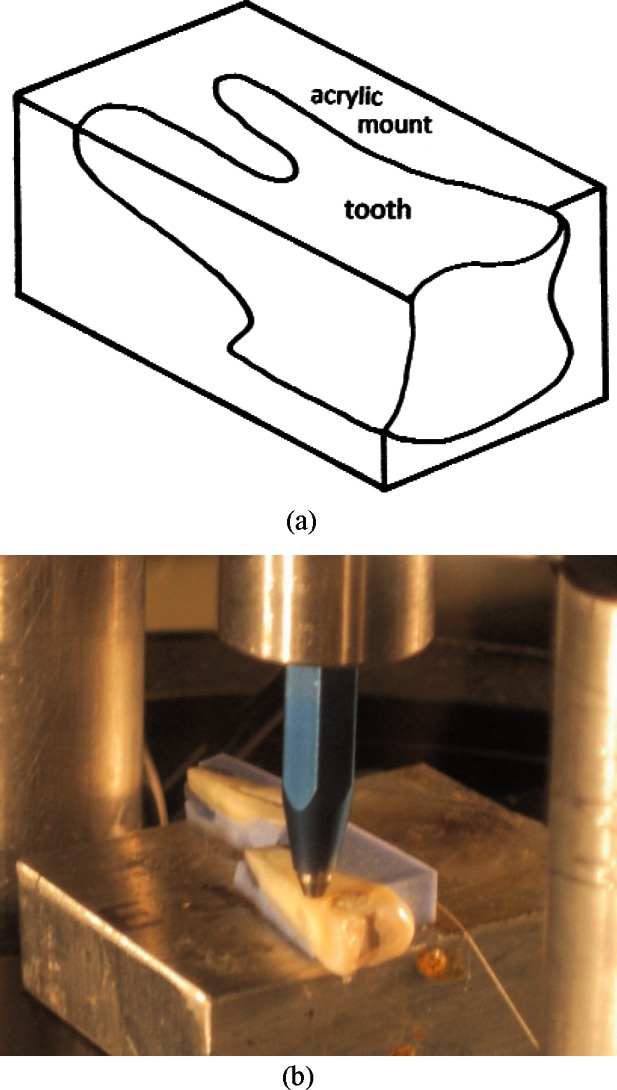
Teeth were mounted in polymethylmethacrylate resin and a 90° edge prepared as shown in (a). Samples were luted to a metal block using machinist’s wax. These were attached to the platform of the edge chipping machine, and the stylus advanced until a chip was produced as shown in (b).

**Fig 3:**
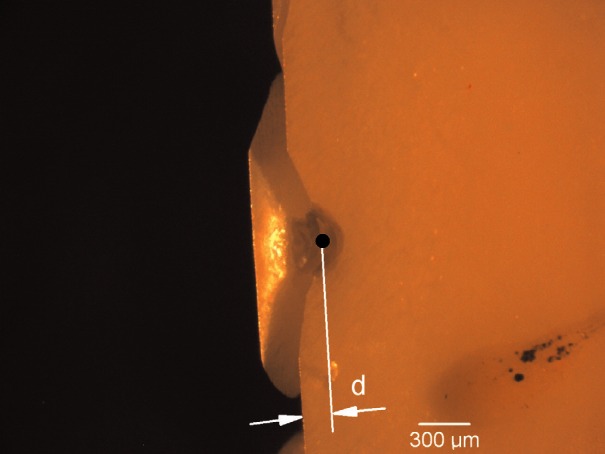
The appearance of a typical edge chip in a view from the top. Note the dent in the tooth structure where the stylus penetrated the surface of the specimen prior to the production of a chip. The edge chip distance was determined microscopically by estimating the distance from the loading point (black dot), to the position of the initial edge.

To prepare teeth for edge chipping, they were embedded in polymethylmethacrylate resin (Plastodent, Inc., Bronx, NY, USA). The acrylic blocks were prepared using a diamond saw (Isomet 1000, Buehler, Lake Bluff, IL, USA) to expose root dentin in the vicinity of the root canals and create the 90 edge required for chipping. Fig. 2a. shows a schematic of the prepared tooth and acrylic block. The exposed dentin surfaces were sequentially polished with 240, 320, 450, and 600 grit sand paper. Each specimen was luted to a metal block using machinist’s wax. Double sided tape was used to secure a prepared specimen-block assembly to the testing platform of an Engineering Systems Model CK 10 edge chipping machine (Nottingham, UK). A conical, diamond scribe indenter (Gilmore Diamond Tools, Attleboro, MA, USA) with a 120° tip angle and a sharp (< 5 μm radius) tip was advanced near the edge of the specimen as shown in Fig. 2b. The load was applied in displacement control with a rate of 1 mm/min and a load cell recorded the force required to produce the chip. Depending on the tooth size, 1 to 5 chips were obtained. Most chips were made in the mid-root region although a few may have been slightly more coronally-located if suitable chips did not form in the mid-root area. In some instances, chips were not formed with the peak indentation load applied since the distance from the edge was too great. A total of thirty five chips in eleven teeth were created. There was insufficient material to obtain valid chips in the 15 day exposed incisor (group 2). A stereo-optical microscope with traveling stage (Model MZ-16, Leica Micro-systems, Wetzlar, Germany) was used to measure the size of the chips to the nearest micrometer as shown in Fig. 3.

For each exposure condition, a plot of chip force versus distance from the edge was created. Regression analysis was performed via least squares with a force-fit through the origin. The slope of this line is the edge toughness, *T_e_*. The standard deviation of the slope of the line (m), and hence the edge toughness, was calculated from the regression analysis using the residual mean square (MSE) and the regression sum of squares (SSR):

s.d.m=s.d.Te=(MSESSR)12m.

A one-way analysis of variance (ANOVA) was used to compare the slopes for each exposure group using a 95 % confidence interval.

## 3. Results

**Fig. 4:**
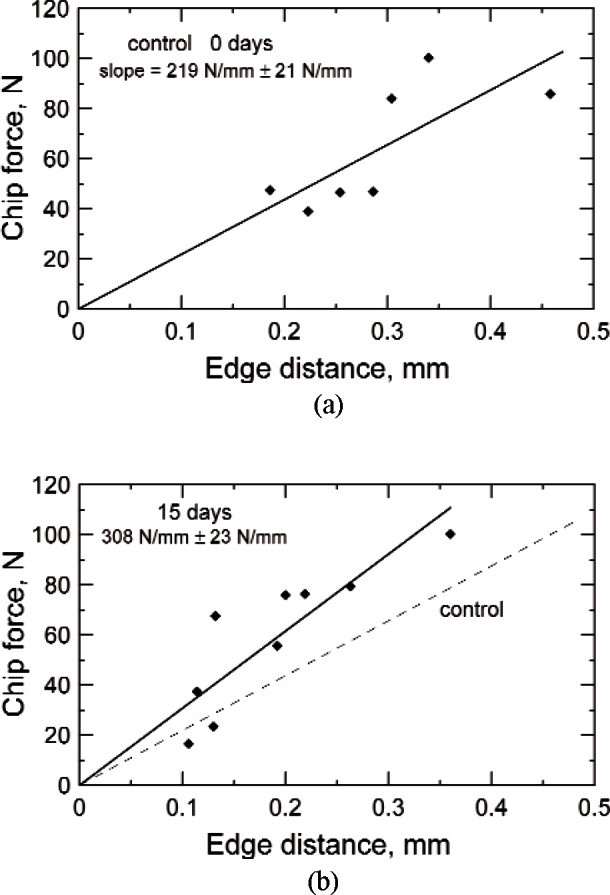

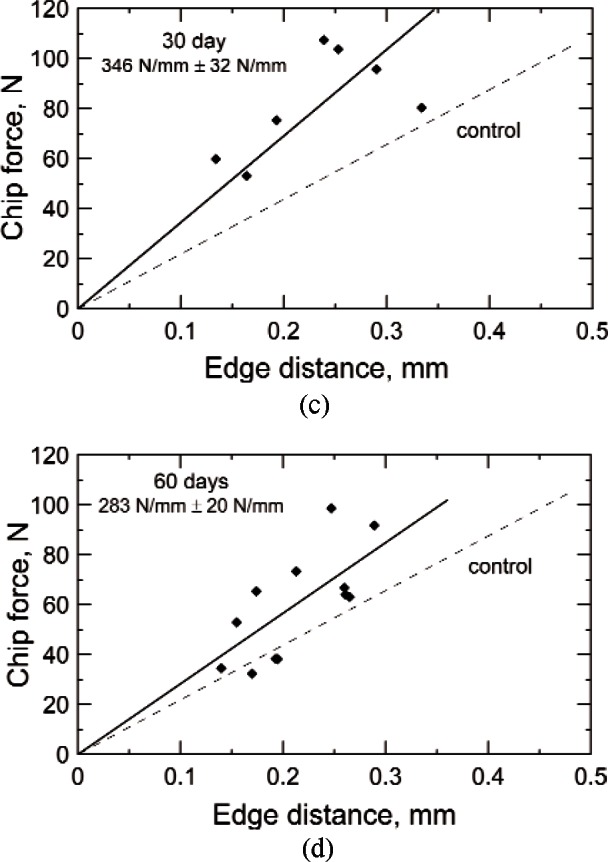
The edge toughness plots for groups 1–4 (a, b, c, d), edge chipped after (zero, 15, 30, and 60) d. Each line demonstrates notable scatter, but all show the trend of increased force required to produce larger chips. The dashed lines are the control data set for 0 d copied from (a). The uncertainties for the line slopes are ± one standard deviation.

Fig. 4. shows the edge toughness plots for each exposure group. While there was high scatter in all the data, an overall trend was evident where a greater force was required to produce larger chips. There did not appear to be a trend among the different tooth types. Some of the variability may be due to variations in the direction of dentinal tubules, the location within the dentin that fracture was initiated, the age of the patient/teeth, and experimental uncertainty, particularly the assessment of the initial contact point. The edge toughness values (± one standard deviation) for groups 1–4, respectively, were 219 (± 21) N/mm, 308 (± 23) N/mm, 346 (± 32) N/mm, and 283 (± 20) N/mm (Fig. 4.). One-way ANOVA showed the calcium hydroxide treated groups’ edge toughness values were significantly different than that for the control group using 95 % confidence intervals. The forces necessary to create edge chips were greater, on average, for the treated specimens than for the untreated controls.

## 4. Discussion

The edge chip test did succeed in causing chip type fractures in dentin. Compared to most dental ceramics and glasses where edge chips are produced with forces in the range of 5 N to 45 N [29], dentin required significantly more force (averaging 45 N to 90 N) before edge chips were formed at distances up to 0.3 mm. This may be due to the greater deformability of dentin relative to ceramics or glasses. Much of the energy of the indentation process may have been used in deformation processes and/or crushing, so that less was available for the formation of a chip fracture. Dentin has a lower elastic modulus and hardness compared to the restoration materials previously tested. The stylus used in this study made a pronounced dent in the surface prior to edge chip formation.

Contrary to the expectation that calcium hydroxide treatment (groups 2–4) would render teeth more susceptible to fracture, the edge chip resistance increased with exposure compared to the untreated controls (group 1). One possible explanation is that the previously cited reports [2, 3, 4] of increased incidence of tooth fracture may pertain to a different mechanism of fracture than chipping. Another possibility is that the change in the exposed specimens may either be a genuine increase in the fracture resistance, or possibly a result of a reduction in dentin hardness. It was noted earlier that edge chip toughness correlates with fracture toughness for many materials, however dentin may be an exception. In most fracture toughness tests, a specimen with a controlled crack is loaded until unstable crack propagation occurs. In an edge chip test on a relatively soft material such as dentin, considerable energy may be expended in deformation processes and in the initiation of a crack at the indentation site. If the calcium hydroxide exposure causes a reduction in dentin hardness, then more energy may be expended in deformation processes than in formation of a chip relative to the control group. In other words, in calcium hydroxide softened dentin, more force may be required to form a chip. A number of studies have shown that calcium hydroxide exposure can reduce hardness measured either by micro or nanoindentation methodologies [9, 34, 35, 36]. These studies show little or no morphological change to the dentin, but the alkalinity of the calcium hydroxide may weaken or damage the collagen tissue. Ceramics and glasses have much higher hardness values than dentin, which should make chipping easier in the latter. The Knoop hardness number (KHN) for dentin is on the order of 68 kg/mm^2^ [37, 38] or varies from (30 to 80) kg/mm^2^ [39]. The hardness of feldspathic porcelain ranges from (460 to 591) kg/mm^2^, depending on the specific formulation [37, 38].

The data in this investigation had considerable scatter on the edge toughness plots as shown in Fig. 4. It is also not known why the 60 d exposed set (group 4) had less edge chip resistance compared to the 15 d and 30 d exposures. It is believed that both the direction of dentinal tubules and age of the patient/teeth influenced edge chipping. Dentin tubule occlusion takes place as teeth age via continued deposition of peritubular dentin [40] which may influence the physical properties of the dentin. Twati et al. [36] and Brännstrom et al. [40] reported partial occlusion of dentin tubules after calcium hydroxide exposures. Likewise, the indenter alignment relative to the axis of the dentinal tubules may also affect chipping behavior. The fracture toughness of dentin is the lowest for crack propagation perpendicular to the tubule axis [41, 42, 43]. It is also well known that dentin properties such as hardness vary with location [39, 44].

## 5. Conclusions

Edge chipping is a feasible test for studying the physical properties of human dentin. All calcium hydroxide exposed test groups showed statistically greater edge toughness values relative to the control group. The edge toughness test, one of many methods to measure fracture resistance, reflects changes in hardness as well as fracture resistance in dentin as a result of the calcium hydroxide exposure. The reported increased incidences of fracture in calcium hydroxide treated teeth may be due to other modes of fracture than chipping.

## Footnotes

1 The views expressed in this article are those of the authors and do not necessarily reflect the official policy or position of the Department of the Navy, Department of Defense, nor the United States Government.

2 Commercial products and equipment are identified only to specify adequately experimental procedures and does not imply endorsement by the authors, the National Institute of Standard and Technology, the National Institute of Health, or the U.S. Navy, nor does it imply that they are necessarily the best for the purpose.

3 NNMC IRB, NNMC.2008.0020.

## References

[1] Nerwich A, Figdor D, Messer HH (1993). pH changes in root dentin over a 4-week period following root canal dressing with calcium hydroxide. J Endodontics.

[2] Rafter M (2005). Apexification: a review, Dent. Traumatology.

[3] Doyon GE, Dumsha T, von Fraunhofer JA (2005). Fracture resistance of human root dentin exposed to intracanal calcium hydroxide. J Endodontics.

[4] Cvek M (1992). Prognosis of luxated non-vital maxillary incisors with calcium hydroxide and filled with gutta percha: a retrospective clinical study. Endod Dent Traumatology.

[5] Pradhan DP, Chawla HS, Gauba K, Goyal A (2006). Comparative evaluation of endodontic management of teeth with unformed apices with mineral trioxide aggregate and calcium hydroxide. J Dent Children.

[6] Andreasen JO, Farik B, Munksgaard EC (2002). Long-term calcium hydroxide as a root canal dressing may increase risk of root fracture. Dent Traumatology.

[7] Tronstad L, Andreasen JO, Hasselgren G, Kristerson L (1981). pH changes in dental tissues after root canal filling with calcium hydroxide. J Endodontics.

[8] Rosenberg B, Murray PE, Namerow K (2007). The effect of calcium hydroxide root filling on dentin fracture strength. Dental Traumatology.

[9] White JD, Lacefield WR, Chavers LS, Eleazer PD (2002). The effect of three commonly used endodontic materials on the strength and hardness of root dentin. J Endodontics.

[10] Grigoratos D, Knowles J, Ng YL, Gulabivala K (2001). Effect of exposing dentine to sodium hypochlorite and calcium hydroxide on its flexural strength and elastic modulus. Int End J.

[11] Siqueira JF, Lopes HP (1999). Mechanisms of antimicrobial activity of calcium hydroxide: a critical review. Int Endodontic J.

[12] Hasselgren G, Olsson B, Cvek M (1998). Effects of calcium hydroxide and sodium hypochlorite on the dissolution of necrotic porcine muscle tissue. J Endodontics.

[13] Andersen M, Lund A, Andreasen JO, Andreasen FM (1992). In vitro solubility of human pulp tissue in calcium hydroxide and sodium hypochlorite. Endod Dent Traumatol.

[14] Hatibovic-Kofman S, Raimundo L, Chong L, Moreno J, Zheng L (2006). Mineral trioxide aggregate in endodontic treatment for immature teeth. Conf Proc IEEE Eng Med Biol Soc.

[15] Hatibovic-Kofman S, Raimundo L, Chong L, Zheng L, Moreno J, Friedman M, Andreasen JO (2008). Fracture resistance and histological findings of immature teeth treated with mineral trioxide aggregate. Dental Traumatology.

[16] Andreasen JO, Munksgaard EC, Bakland LK (2006). Comparison of fracture resistance in root canals of immature sheep teeth after filling with calcium hydroxide or MTA. Dental Traumatology.

[17] Andreasen JO, Farik B, Munksgaard EC (2002). Long-term calcium hydroxide as a root canal dressing may increase risk of root fracture. Dental Traumatology.

[18] Kelly JR (2004). Dental ceramics: current thinking and trends. Dent Clin N America.

[19] Kishen A (2006). Mechanisms and risk factors for fracture predilection in endodontically treated teeth. Endodontic Topics.

[20] Almond EA, McCormick NJ (1986). Constant-geometry edge-flaking of brittle materials. Nature.

[21] McCormick NJ (1992). Edge flaking as a measure of material performance. Metals and Materials.

[22] Quinn JB, Lloyd IK, Varner JR, Quinn GD (2001). Flake and scratch size ratios in ceramics. Fractography of Glasses and Ceramics IV, Ceramic Transactions 122.

[23] Quinn JB, Ram M (2005). Vaderhobli, Geometry of edge chips formed at different angles, Ceram. Eng Sci Proc.

[24] Danzer R, Hangl M, Paar R (2001). Edge Chipping of Brittle Materials. Fractography of Glasses and Ceramics IV.

[25] Quinn GD (2007). Guide to practice for fractography of ceramics and glasses.

[26] Quinn JB, Hatch JW, Bradt RC, Varner JR, Quinn GD (2001). The edge flaking test as an assessment of the thermal alteration of lithic materials, Bald Eagle jasper. Fractography of Glasses and Ceramics IV.

[27] Gogotsi G, Mudrik S, Galenko V (2007). Evaluation of Fracture Resistance of Ceramics: Edge Fracture Tests. Ceram Int.

[28] Gogotsi G, Mudrik S (2009). Fracture Barrier Estimation by the Edge Fracture Test Method. Ceram Int.

[29] Quinn JB, Su L, Flanders L, Lloyd IK (2000). “Edge toughnes” and material properties related to the machining of dental ceramics. Mach Sci and Technol.

[30] Quinn JB, Sundar V, Parry EE, Quinn GD (2010). Comparison of edge chipping resistance of PFM and veneered zirconia specimens. Dent Mater.

[31] Watts DC, Issa M, Ibrahim A, Wakiga J, Al-Samadani K, Al-Azraqi M, Silikas N (2008). Edge Strength of Resin-composite margins. Dent Mat.

[32] Baroudi K, Silikas N, Watts DC (2008). Edge-strength of flowable resin-composites. J Dentistry.

[33] Fava LRG, Saunders WP (1999). Calcium hydroxide pastes: classification and clinical indications. Int Endodontic J.

[34] Yoldas O, Dogan C, Seydaoglu G (2004). The effect of two different calcium hydroxide combinations on root dentine microhardness. Int Endodontic J.

[35] Tsuzuki T, Ogawa H, Kitamura K, Yamazaki T, Katsuumi I, Koushi R, Akutsu K, Sato N, Oida S (2000). A study on hardness change of root canal dentin applied with calcium hydroxide. Jap J Conserv Dent.

[36] Twati WA, Wood DJ, Liskiewicz TW, Willmott NS, Duggal MS (2009). An evaluation of the effect of non-setting calcium hydroxide on human dentine: a pilot study. Eur Arch Paediatr Dent.

[37] Craig RG, Powers JM (2002). Restorative dental materials.

[38] O’Brien WJ (2002). Dental Materials and their selection.

[39] Pashley D, Okabe A, Parham P (1985). The relationship between dentin microhardness and tubule density. Endod Dent Traumat.

[40] Brännstrom M, Isacsson G, Johnson G (1976). The effect of calcium hydroxide and fluorides on human dentine. Acta Odont.

[41] Imbeni V, Nalla RK, Bosi C, Kinney JH, Ritchie RO (2003). On the in vitro fracture toughness of human dentin. J Biomed Mater Res A.

[42] Iwamoto N, Ruse ND (2003). Fracture toughness of human dentin. J Biomed Mater Res A.

[43] Arola D, Ivancik J, Majd H, Bajaj D, Zhang X, Fouad AF (2011). On the Microstructure and Mechanical Behavior of Radicular and Coronal Dentin, to be publ. Endodontic Topics.

[44] Kinney JH, Balooch M, Marshall SJ, Marshall GW, Weihs TP (1996). Hardness and Young’s modulus of human peritubular and intertubular dentine. Archs Oral Biol.

